# Knowledge, attitude, and practice toward sleep disorders and sleep hygiene among perimenopausal women

**DOI:** 10.1038/s41598-024-62502-4

**Published:** 2024-05-22

**Authors:** Xiaomin Shi, Yi Shi, Jie Wang, Hui Wang, Yunru Li

**Affiliations:** 1 Dongzhimen Hospital, Beijing University of Chinese Medicine, Beijing, 100700 China; 2 Ningjin TCM Hospital, Shandong, 253400 China; 3 Beijing University of Chinese Medicine, Beijing, 100105 China

**Keywords:** Knowledge, Attitude, Practice, Sleep disorders, Sleep hygiene, Perimenopausal women, Public health, Circadian rhythms and sleep

## Abstract

This cross-sectional study aimed to explore the knowledge, attitude and practice (KAP) toward sleep disorders and sleep hygiene among perimenopausal women, who were enrolled in Dezhou region of Shandong Province between July and September 2023. A total of 720 valid questionnaires were collected (mean age: 51.28 ± 4.32 years old), and 344 (47.78%) reported experiencing insomnia. The mean scores for knowledge, attitude, practice, and Dysfunctional Beliefs and Attitudes about Sleep (DBAS) were 15.73 ± 7.60 (possible range: 0–36), 29.35 ± 3.15 (possible range: 10–50), 28.54 ± 4.03 (possible range: 10–50), and 6.79 ± 1.90 (possible range: 0–10), respectively. Path analysis showed that knowledge had direct effects on attitude (*β* = 0.04, 95% CI 0.01–0.07, *P* = 0.001), and DBAS (*β* = 0.04, 95% CI 0.02–0.05, *P* < 0.001). Knowledge had direct effects (*β* = 0.11, 95% CI 0.08–0.15, *P* < 0.001) and indirect (*β* = 0.02, 95% CI 0.00–0.03, *P* = 0.002) effect on practice. Moreover, attitude also had a direct impact on practice (*β* = 0.34, 95% CI 0.25–0.43, *P* < 0.001). In conclusion, perimenopausal women exhibited insufficient knowledge, negative attitude, inactive practice toward sleep disorders and sleep hygiene, and unfavorable DBAS, emphasizing the need for targeted healthcare interventions.

## Introduction

Sleep disorders, stemming from factors such as stress, anxiety, poor sleep hygiene, and daytime overexcitation, affect individuals, particularly women in various stages of menopause. Studies reveal a prevalence of sleep disorders ranging from 16 to 42% in premenopausal women, 39–47% in perimenopausal women, and 35% to 60% in postmenopausal women^1–3^. The transition to menopause introduces physiological and hormonal changes that disrupt sleep patterns, contributing to a spectrum of sleep-related challenges^4,5^. Sleep disturbances present potential hazards to the physiological and psychological well-being of perimenopausal women. Acknowledging the potential implications for their overall health, it is imperative to undertake a thorough exploration of the sleep patterns specific to perimenopausal women. Recognizing the importance of addressing these issues, sleep hygiene emerges as a supportive strategy to enhance sleep quality in menopausal women^3^. Sleep hygiene recommendations, encompassing behavioral and environmental instructions, aim to promote sleep onset, maintenance, and overall quality^6^. It is crucial to understand the nuances of sleep disorders and hygiene in perimenopausal women for effective healthcare strategies, as adequate sleep is integral to overall well-being^7,8^.

Knowledge, Attitude, and Practice (KAP) studies are quantitative research methods aimed at assessing individuals' understanding, attitude, and behavior related to a specific topic, providing insights for targeted interventions and informed decision-making in fields such as health, social sciences, and education^9–11^. This model helps explain why perimenopausal women might not seek timely medical help for sleep disorders. Many women mistakenly consider sleep problems as a normal part of menopause rather than a treatable health issue. The lack of sufficient knowledge about sleep disorders likely hinders their understanding of available treatment options, leading to unnecessary discomfort. Research on sleep disorders and sleep hygiene among perimenopausal women is clinically significant as it sheds light on the specific challenges faced by this demographic during a critical life transition. The perimenopausal phase, characterized by hormonal fluctuations, often leads to sleep disorders, impacting mental health and overall well-being^12^. Therefore, investigating sleep hygiene provides insights into daily sleep habits, laying the groundwork for effective health education and behavioral interventions. By promoting healthy sleep practice, we not only alleviate sleep issues but also positively contribute to overall health maintenance. The research outcomes can inform healthcare institutions, optimizing resources and enhancing attention and management for perimenopausal women. Therefore, this study aimed to explore the KAP toward sleep disorders and sleep hygiene among perimenopausal women.

## Results

### Basic characteristics

A total of 918 questionnaires were initially received, with 93 cases excluded for an answering time less than 120 s, 100 cases for age less than 45 or greater than 60, and 31 cases where all options for each question in the KAP section were consistent, resulting in a total of 720 valid questionnaires (78.43%). Among these perimenopausal women, the mean age was 51.28 ± 4.32 years old, 692 (96.11) were married, 351 (48.75%) lived in rural areas, 326 (45.28%) of them had more education than Junior high school, 506 (70.28%) people's average monthly per capita income is less than 5000 RMB, with an average sleep duration of 7.12 ± 1.36 h per day, and 119 (16.53%) of them falling asleep after 23:00. Furthermore, 344 (47.78%) reported experiencing insomnia, 352 (48.89%) exhibited symptoms related to perimenopausal syndrome, 109 (15.14%) experienced menstrual irregularities, 174 (24.17%) experienced hot flashes, night sweats, and 217 (30.14%) presented with emotional instability, anxiety, depression, or irritability (Table 1).

**Table 1:** Baseline characteristics and knowledge, attitude and practice scores.

Variables	N (%)	Knowledge Score		Attitude Score		Practice Score		DBAS-16 Score	
		Mean ± SD	P	Mean ± SD	P	Mean ± SD	P	Mean ± SD	P
*N* = 720									
Total Score		15.73 ± 7.60		29.35 ± 3.15		28.54 ± 4.03		6.79 ± 1.90	
Age (years)	51.28 ± 4.32								
Marital status									
Single*	28 (3.89)	16.35 ± 9.27	0.674	29.53 ± 3.27	0.649	27.46 ± 4.29	0.103	6.79 ± 1.60	0.867
Married	692 (96.11)	15.70 ± 7.53		29.34 ± 3.15		28.58 ± 4.01		6.78 ± 1.90	
Residence									
Rural	351 (48.75)	15.38 ± 7.77	0.293	28.92 ± 2.99	0.002	28.39 ± 4.10	0.030	6.79 ± 1.88	0.422
Urban	299 (41.53)	16.16 ± 7.52		29.84 ± 3.26		28.97 ± 3.95		6.85 ± 1.84	
Suburban	70 (9.72)	15.57 ± 7.09		29.37 ± 3.14		27.38 ± 3.70		6.44 ± 2.17	
Education									
Primary school and below	100 (13.89)	13 ± 6.46	< 0.001	28.6 ± 2.88	< 0.001	27.29 ± 4.07	< 0.001	6.65 ± 1.67	0.612
Junior high school	294 (40.83)	15.55 ± 7.49		28.96 ± 3.02		28.24 ± 4.05		6.82 ± 1.89	
High school/Technical secondary school	166 (23.06)	15.98 ± 8.28		29.83 ± 3.32		28.95 ± 3.66		6.79 ± 2.02	
College/bachelor's/master's degree	160 (22.22)	17.47 ± 7.26		30.03 ± 3.17		29.44 ± 4.08		6.79 ± 1.90	
Average monthly per capita income (RMB)									
< 2000	166 (23.06)	14.36 ± 7.49	0.004	28.68 ± 3.30	< 0.001	27.90 ± 4.11	0.077	6.70 ± 1.88	0.498
2000–5000	340 (47.22)	15.53 ± 7.58		29.31 ± 3.04		28.50 ± 3.91		6.78 ± 1.86	
5000–10,000	166 (23.06)	17.31 ± 7.62		30.13 ± 3.07		29.19 ± 4.08		6.75 ± 2.01	
> 10,000	48 (6.67)	16.33 ± 7.18		29.22 ± 3.17		28.72 ± 4.07		7.13 ± 1.77	
Average sleep hours per day	7.12 ± 1.36								
Time to go to sleep									
Before 22:00	252 (35)	16.20 ± 7.91	0.286	29.28 ± 2.77	0.219	29.05 ± 4.06	< 0.001	7.04 ± 1.81	0.019
22:01–23:00	349 (48.47)	15.64 ± 7.40		29.59 ± 3.24		28.62 ± 3.87		6.70 ± 1.89	
After 23:00	119 (16.53)	14.94 ± 7.51		28.78 ± 3.53		27.21 ± 4.14		6.47 ± 2.01	
Insomnia									
Yes	344 (47.78)	15.28 ± 7.06	0.159	29.07 ± 3.35	0.060	28.22 ± 4.05	0.043	6.70 ± 1.82	0.343
No	376 (52.22)	16.13 ± 8.05		29.60 ± 2.93		28.82 ± 3.98		6.86 ± 1.95	
Symptoms related to perimenopausal syndrome									
Yes	352 (48.89)	16.06 ± 7.86	0.326	29.25 ± 3.21	0.189	28.48 ± 3.95	0.561	6.80 ± 1.95	0.734
No	368 (51.11)	15.37 ± 7.31		29.45 ± 3.08		28.59 ± 4.11		6.76 ± 1.84	
Symptoms related to perimenopausal syndrome (Multiple choices)									
None			368 (51.11)						
Menstrual irregularities			109 (15.14)						
Hot flashes, night sweats			174 (24.17)						
Emotional instability, anxiety, depression, or irritability			217 (30.14)						
Other			17 (2.36)						

* Single includes unmarried, divorced and widowed.

### Dimensions of knowledge, attitude, practice

The mean knowledge, attitude, practice, and DBAS-16 scores were 15.73 ± 7.60 (possible range: 0–36), 29.35 ± 3.15 (possible range: 10–50), 28.54 ± 4.03 (possible range: 10–50), and 6.79 ± 1.90 (possible range: 0–10) respectively. There were significant differences in knowledge scores toward sleep disorders and sleep hygiene among people with different education (*P* < 0.001) and income levels (*P* = 0.004). For the attitude scores, significant differences were observed in residence (*P* = 0.002), educational levels (*P* < 0.001), and people's per capita household income (*P* < 0.001). For the practice scores, significant differences were observed in residence (*P* = 0.030), education (*P* < 0.001), time to sleep (*P* < 0.001), insomnia experience (*P* = 0.043). There were significant differences in DBAS-16 scores in time to sleep (*P* = 0.019) (Table 1). Analysis of demographic characteristics and DBAS-16 scores revealed that participants going to bed before 22:00 were more likely to have higher DBAS-16 consequence scores (*P* = 0.016), expectation scores (*P* = 0.020), medication scores (*P* = 0.045), and total scores (*P* = 0.019). Those from urban areas (*P* = 0.009), those with higher education (*P* = 0.045), and those without insomnia (*P* < 0.001) were more likely to have higher DBAS-16 expectation scores (Supplementary Table 1).

Responses to the knowledge dimension showed that more than 60% of the participants identified decreased sleep quality (K1.2), a total sleep duration of less than 8 h (K1.3), and daytime dysfunction (K1.4) as the main symptoms of insomnia (Supplementary Table 2). The highest proportion of participants choosing the “Aware” option were “Research indicates that the optimal bedtime for adults is between 22:00 and 23:00, with an ideal sleep duration of 7–8 h” (K10) with 35% (Supplementary Table 2). Participants' attitude showed that 39.72% believed that perimenopausal sleep disorders are quite normal and do not need special treatment (A1), 72.36% said that nocturnal sleep disorders seriously interfere with my daytime life (A3), and 47.08% strongly agreed that creating a comfortable sleep environment can help improve sleep quality (A6). 35.14% were very willing to participate to perimenopausal sleep disorders related educational lectures (A10) (Supplementary Table 3). Responses to the practice dimension revealed a variety of behavioral habits among participants, with 64.3% always or often having a regular daily schedule (P1). Respectively, 59.31% and 59.17% reported that they never use alcohol (P2) or sleeping pills (P3) to help them fall asleep. In addition, when the insomnia problem was serious, 32.78% sometimes actively seek medical advice from a doctor (P10) (Supplementary Table 4).

### Person correlation analysis

Person correlation analysis revealed a positive correlation of knowledge with attitude (*r* = 0.174, *P* < 0.001), practice (*r* = 0.276, *P* < 0.001), and DBAS (*r* = 0.169, *P* < 0.001). Attitude was positively correlated with practice (*r* = 0.312, *P* < 0.001) (Table 2).

**Table 2:** Pearson correlation Analysis.

	Knowledge	Attitude	Practice	DBAS
Knowledge	1			
Attitude	0.174 (*P* < 0.001)	1		
Practice	0.276 (*P* < 0.001)	0.312 (*P* < 0.001)	1	
DBAS	0.169 (*P* < 0.001)	-0.010 (*P* = 0.796)	0.058 (*P* = 0.118)	1

### Multivariate logistic regression analysis

Multivariate logistic regression analysis showed that DBAS (O*r* = 1.33, 95% CI: 1.19–1.48, *P* < 0.001) and education of “Junior high school” (O*r* = 2.81, 95% CI: 1.27–6.20, *P* = 0.011), “High school/Technical secondary school” (O*r* = 3.16, 95% CI: 1.37–7.31, *P* = 0.007), and “College/bachelor's/master's degree” (O*r* = 4.22, 95% CI: 1.79–9.94, *P* = 0.001) were independently associated with knowledge. Also, knowledge score (O*r* = 1.08, 95% CI: 1.05–1.10, *P* < 0.001), “High school/Technical secondary school” (O*r* = 1.99, 95% CI: 1.10–3.61, *P* = 0.022), and average monthly per capita income of 5000–10,000 RMB (O*r* = 1.79, 95% CI: 1.00–3.21, *P* = 0.049) were independently associated with attitude. Moreover, knowledge score (O*r* = 1.05, 95% CI: 1.03–1.07, *P* < 0.001), attitude score (O*r* = 1.18, 95% CI: 1.11–1.25, *P* < 0.001), “College/bachelor's/master's degree” (O*r* = 1.96, 95% CI: 1.10–3.49, *P* = 0.021), longer average time of sleep per day (O*r* = 1.15, 95% CI: 1.01–1.30, *P* = 0.028), and sleeping after 23:00 (O*r* = 0.44, 95% CI: 0.26–0.73, *P* = 0.002) were independently associated with practice (Table 3).

**Table 3:** Univariate and multivariate logistic regression analysis.

Dimensions	Variables	Univariate analysis			Multivariate analysis	P
		OR (95% CI)		P	OR (95%CI)	
Knowledge						
	DBAS	1.35 (1.21, 1.50)		< 0.001	1.33 (1.19, 1.48)	< 0.001
	Age (years)	0.95 (0.91, 0.99)		0.044	0.97 (0.93, 1.02)	0.301
	Marital Status					
	Single*	Ref				
	Married	0.68 (0.29, 1.58)		0.379		
	Residence					
	Rural	Ref				
	Urban	1.04 (0.72, 1.51)		0.817		
	Suburban	0.90 (0.47, 1.71)		0.758		
	Education					
	Primary school and below	Ref			Ref	
	Junior high school	3.01 (1.38, 6.53)		0.005	2.81 (1.27, 6.20)	0.011
	High school/Technical secondary school	3.65 (1.63, 8.16)		0.002	3.16 (1.37, 7.31)	0.007
	College/bachelor's/master's degree	4.92 (2.21, 10.9)		< 0.001	4.22 (1.79, 9.94)	0.001
	Average monthly per capita income (RMB)					
	< 2000	Ref			Ref	
	2000–5000	1.25 (0.77, 2.03)		0.359	1.05 (0.62, 1.76)	0.850
	5000–10,000	2.00 (1.18, 3.39)		0.010	1.53 (0.84, 2.77)	0.160
	> 10,000	1.64 (0.76, 3.54)		0.206	1.14 (0.49, 2.61)	0.753
	Average sleep hours per day	1.13 (0.99, 1.28)		0.059		
	Time to go to sleep					
	Before 22:00	Ref			Ref	
	22:01–23:00	0.66 (0.45, 0.98)		0.042	0.67 (0.44, 1.02)	0.064
	After 23:00	0.71 (0.41, 1.20)		0.208	0.72 (0.40, 1.28)	0.266
	Insomnia					
	Yes	Ref			Ref	
	No	0.62 (0.43, 0.90)		0.012	0.71 (0.48, 1.05)	0.093
	Symptoms related to perimenopausal syndrome					
	Yes	Ref				
	No	0.80 (0.56, 1.14)		0.223		
	Variables		Univariate Analysis		Multivariate Analysis	
			OR (95%CI)	P	OR (95%CI)	P
Attitude						
	Knowledge		1.06 (1.04, 1.09)	< 0.001	1.08 (1.05, 1.10)	< 0.001
	DBAS		1.11 (1.01, 1.21)	0.02	1.07 (0.97, 1.18)	0.136
	Age (years)		0.98 (0.94, 1.01)	0.303		
	Marital Status					
	Single*		Ref			
	Married		0.76 (0.30, 1.91)	0.566		
	Residence					
	Rural		Ref			
	Urban		1.37 (0.96, 1.97)	0.078		
	Suburban		0.92 (0.53, 1.62)	0.799		
	Education					
	Primary school and below		Ref		Ref	
	Junior high school		1.53 (0.95, 2.46)	0.079	1.34 (0.81, 2.22)	0.246
	High school/Technical secondary school		2.56 (1.46, 4.48)	0.001	1.99 (1.10, 3.61)	0.022
	College/bachelor's/master's degree		2.27 (1.30, 3.95)	0.004	1.54 (0.82, 2.87)	0.171
	Average monthly per capita income (RMB)					
	< 2000		Ref		Ref	
	2000–5000		1.39 (0.93, 2.09)	0.104	1.20 (0.77, 1.85)	0.404
	5000–10,000		2.34 (1.39, 3.91)	0.001	1.79 (1.00, 3.21)	0.049
	> 10,000		1.09 (0.54, 2.17)	0.807	0.81 (0.37, 1.73)	0.591
	Average sleep hours per day		1.03 (0.91, 1.17)	0.589		
	Time to go to sleep					
	Before 22:00		Ref			
	22:01–23:00		1.10 (0.75, 1.59)	0.609		
	After 23:00		0.78 (0.48, 1.26)	0.326		
	Insomnia					
	Yes		Ref			
	No		0.75 (0.53, 1.05)	0.102		
	Symptoms related to perimenopausal syndrome					
	Yes		Ref			
	No		1.32 (0.94, 1.85)	0.103		
	Variables		Univariate Analysis		Multivariate Analysis	
			OR (95%CI)	P	OR (95%CI)	P
Practice						
	Knowledge score		1.06 (1.04, 1.08)	< 0.001	1.05 (1.03, 1.07)	< 0.001
	Attitude score		1.21 (1.14, 1.28)	< 0.001	1.18 (1.11, 1.25)	< 0.001
	DBAS		1.05 (0.97, 1.14)	0.152		
	Age (years)		0.95 (0.92, 0.99)	0.011	0.96 (0.93, 1.00)	0.094
	Marital Status					
	Single*		Ref			
	Married		1.71 (0.79, 3.67)	0.168		
	Residence					
	Rural		Ref			
	Urban		1.24 (0.91, 1.69)	0.171		
	Suburban		0.85 (0.51, 1.43)	0.556		
	Education					
	Primary school and below		Ref		Ref	
	Junior high school		1.81 (1.14, 2.88)	0.011	1.50 (0.91, 2.47)	0.104
	High school/Technical secondary school		2.05 (1.24, 3.40)	0.005	1.47 (0.85, 2.54)	0.164
	College/bachelor's/master's degree		2.78 (1.66, 4.66)	< 0.001	1.96 (1.10, 3.49)	0.021
	Average monthly per capita income (RMB)					
	< 2000		Ref			
	2000–5000		1.17 (0.81, 1.70)	0.388		
	5000–10,000		1.34 (0.86, 2.06)	0.186		
	> 10,000		1.19 (0.62, 2.28)	0.587		
	Average sleep hours per day		1.21 (1.08, 1.37)	0.001	1.15 (1.01, 1.30)	0.028
	Time to go to sleep each day					
	Before 22:00		Ref		Ref	
	22:01–23:00		0.78 (0.56, 1.09)	0.159	0.75 (0.52, 1.09)	0.138
	After 23:00		0.43 (0.27, 0.67)	< 0.001	0.44 (0.26, 0.73)	0.002
	Insomnia					
	Yes		Ref			
	No		0.86 (0.64, 1.15)	0.322		
	Symptoms related to perimenopausal syndrome					
	Yes		Ref			
	No		1.11 (0.83, 1.50)	0.457		

* Single includes unmarried, divorced and widowed.

### Path analysis

Path analysis showed that knowledge had direct effect on attitude (*β* = 0.04, 95% CI: 0.01–0.07, *P* = 0.001), and DBAS (*β* = 0.04, 95% CI: 0.02–0.05, *P* < 0.001). Knowledge had direct effect (*β* = 0.11, 95% CI: 0.08–0.15, *P* < 0.001) and indirect (*β* = 0.02, 95% CI: 0.00–0.03, *P* = 0.002) effect on practice. Moreover, attitude also have a direct impact on practice (*β* = 0.34, 95% CI: 0.25–0.43, *P* < 0.001) (Table 4).

**Table 4:** Path analysis.

Model paths		Total effects		Direct Effect		Indirect effect	
		β (95% CI)	P	β (95% CI)	P	β (95% CI)	P
Attitude < -							
	Knowledge	0.04 (0.01, 0.07)	0.001	0.04 (0.01, 0.07)	0.001		
Practice < -							
	Attitude	0.34 (0.25, 0.43)	< 0.001	0.34 (0.25, 0.43)	< 0.001		
	DBAS	0.07(-0.0, 0.21)	0.327	0.07 (-0.0, 0.21)	0.327		
	Knowledge	0.13 (0.09, 0.17)	< 0.001	0.11 (0.08, 0.15)	< 0.001	0.02 (0.00, 0.03)	0.002
DBAS < -							
	Knowledge	0.04 (0.02, 0.05)	< 0.001	0.04 (0.02, 0.05)	< 0.001		

## Discussion

Perimenopausal women had inadequate knowledge, negative attitude, inactive practice, and unfavorable DBAS toward sleep disorders and sleep hygiene. The research findings from this study provide valuable insights into sleep disorders and sleep hygiene among perimenopausal women.

This study indicated a concerning lack of awareness and engagement with positive sleep-related behaviors among perimenopausal women, which aligns with existing literature emphasizing the vulnerability of perimenopausal women to sleep disorders due to hormonal changes and associated symptoms^13,14^. This study also found that residence emerged as a significant factor, with urban residents showing higher mean knowledge and practice scores compared to their rural counterparts, which may be attributed to variations in access to information, healthcare resources, and lifestyle patterns between urban and rural environments^15,16^, underscoring the need for targeted interventions tailored to the specific needs of different populations. Educational level emerged as a robust predictor of sleep related KAP scores, with higher education associated with more favorable outcomes. This finding was consistent with existing research highlighting the positive correlation between education and health literacy, suggesting that well-educated individuals are more likely to adopt healthier behaviors and seek relevant information^17,18^. The influence of income levels on KAP scores, particularly the elevated scores in the highest income group (> 10,000 RMB), warrants further investigation. It may be indicative of better access to healthcare resources. Sleep-related practice, such as the time to sleep, demonstrated noteworthy associations with knowledge and practice. Participants who going to sleep after 23:00 exhibited negative practice compared to those slept before 23:00. This aligns with established sleep hygiene recommendations that emphasize the importance of consistent and earlier sleep patterns for optimal sleep quality^19,20^. The impact of insomnia on knowledge and practice scores further underscores the need for targeted interventions for individuals experiencing sleep disorders.

Pearson correlation analysis revealed positive correlations between knowledge, attitude, practice, and DBAS scores, indicating that these factors are interconnected. This interrelation emphasizes the need for comprehensive interventions that target multiple dimensions simultaneously, acknowledging the intricate relationship between knowledge, attitude, and practice toward sleep disorders. Multivariate logistic regression analysis found that DBAS and higher education were identified as independent factors associated with knowledge. Similarly, education, income, and knowledge were independently associated with attitude. Notably, proactive practice was associated with higher knowledge and attitude scores, emphasizing the importance of addressing both cognitive and attitudinal aspects in promoting positive sleep-related behaviors. Sleeping after 23:00 was associated with negative practice, reinforcing the significance of sleep timing in influencing sleep-related practice among perimenopausal women. Path analysis indicated direct effects of knowledge on attitude, practice, and DBAS, reinforcing the pivotal role of knowledge in shaping attitude and behaviors related to sleep. The direct impact of attitude on practice highlighted the mediating role of attitude in the knowledge-practice pathway. These findings underscore the need for targeted and multifaceted interventions to improve the sleep-related knowledge, attitude, and practice among perimenopausal women^21,22^. These interventions should consider demographic factors such as residence, education, and income levels to tailor strategies effectively. Addressing negative sleep-related practice, particularly related to sleep timing, is crucial in promoting positive sleep behaviors^23,24^.

For knowledge dimension, it is noteworthy that participants exhibited disparities in their awareness of insomnia-related concepts. The highest-scoring item, indicating awareness of the optimal bedtime for adults and the recommended sleep duration, reflects a substantial understanding among participants. On the contrary, the lowest-scoring item, pertaining to the belief that alcohol consumption before bedtime improves sleep quality, reveals a prevalent misconception. Addressing this specific knowledge gap is crucial, as it influences individuals' choices and behaviors. To improve knowledge toward the efficacy of alcohol on sleep quality, interventions should debunk common myths through targeted educational campaigns^25–27^. Collaborative efforts with healthcare professionals and community organizations can facilitate the dissemination of evidence-based information. Practical and accessible resources, such as informational leaflets or online modules, can serve as valuable tools in challenging and reshaping misconceptions^28^.

For attitude dimension, participants' responses reveal varying perspectives on perimenopausal sleep disorders. The item indicating a willingness to participate in educational lectures on sleep disorders, reflects a positive attitude toward seeking knowledge and support. Conversely, the item reflecting the belief that sleep disorders during perimenopause are normal and do not require special treatment, signifies a potentially detrimental attitude. This perception may hinder timely interventions and exacerbate the impact of sleep disorders on overall well-being. To address this attitude, interventions should target misconceptions about the normalcy of sleep disorders during perimenopause^29,30^. Educational campaigns can incorporate narratives from individuals who have successfully managed sleep issues during this life stage, emphasizing the benefits of seeking professional guidance. Psychosocial support groups or forums could provide platforms for shared experiences, fostering a more nuanced understanding of perimenopausal sleep disorders.

For practice dimension, participants' responses reveal a variety of sleep-related behaviors. The item that gauges the consistency of participants' daily schedules highlights a favorable practice linked with sleep hygiene. Conversely, the item reflecting involvement in stimulating activities before bedtime underscores potentially counterproductive practice that may contribute to poor sleep hygiene. Such behaviors are recognized as risk factors for insomnia and may warrant targeted interventions. To address suboptimal practice, interventions should emphasize the importance of sleep hygiene and provide practical strategies to enhance sleep-related behaviors. Behavioral interventions, such as stimulus control therapy, can be introduced to modify practice associated with poor sleep hygiene^31^. Educational materials should be tailored to address specific problematic practice identified in the study, promoting realistic and feasible alternatives that align with evidence-based recommendations. In light of our results, it would be advisable for policymakers to consider augmenting current sleep guidelines with additional educational content specifically aimed at perimenopausal women. Future policies might include the development of specific sleep education modules for perimenopausal women as part of regular health check-ups, thereby institutionalizing a more proactive approach to sleep disorder management in this vulnerable group.

Several measures could be considered to address the sleep-related issues among perimenopausal women. Firstly, updating the existing sleep guidelines in China to include specific recommendations for perimenopausal women would be crucial. These guidelines should provide detailed explanations about sleep disorders and hygiene tailored to this demographic, facilitating the adoption of positive sleep behaviors. Secondly, implementing sleep education programs targeting perimenopausal women could be beneficial. These programs could include workshops, lectures, or online courses aimed at providing relevant knowledge about sleep disorders and hygiene and promoting positive sleep practices. Thirdly, providing training for healthcare professionals in identifying and managing sleep disorders and hygiene issues among perimenopausal women would be essential. This would ensure that healthcare providers are equipped to offer appropriate support and guidance. Additionally, community-based intervention projects targeting perimenopausal women could be established. These projects could involve setting up sleep health education centers, offering sleep health counseling services, and providing support groups to enhance sleep health awareness and support. Lastly, supporting and encouraging research initiatives focusing on sleep issues among perimenopausal women would be crucial. These initiatives could help in understanding the underlying causes and factors influencing sleep problems and exploring effective intervention strategies. Through these policy measures, it is possible to improve awareness of sleep-related issues among perimenopausal women, promote positive sleep behaviors, and ultimately enhance their sleep quality and overall well-being.

The strengths of this study included its robust sample size, rigorous data collection methods, and the utilization of a path analysis, providing valuable insights into the complex relationships among knowledge, attitude, and practice on sleep disorders and sleep hygiene among perimenopausal women. However, there were still several limitations in this study. Finally, the data relied on self-reported data, which may be subject to recall bias. Secondly, this is a cross-sectional study, and the causal relationship cannot be determined. Thirdly, this study was restricted to the Dezhou region, Shandong, which may limit its generalizability to other area.

In conclusion, perimenopausal women demonstrated insufficient knowledge, negative attitude, inactive practice toward sleep disorders and sleep hygiene, and unfavorable DBAS, which underscore the critical need for targeted educational interventions aimed at improving knowledge and fostering positive attitude and practice related to sleep disorders and hygiene among perimenopausal women.

## Methods

### Study design and participants

This cross-sectional study, conducted between July and September 2023, gathered data from perimenopausal women at the Dezhou region of Shandong Province. Inclusion criteria: (1) females aged 40–60 years; (2) understanding the questionnaire without communication barriers; (3) volunteered to participate in this study. Exclusion criteria: (1) presence of conditions such as work- or environment-related insomnia; (2) recent use of corticosteroids, systemic illnesses, or substance abuse; (3) cognitive disorders; (4) pregnancy or lactation. The study was carried out after the protocol was approved by the Ethics Committee of Dongzhimen Hospital Affiliated to Beijing University of Chinese Medicine(2023DZMEC-174–01). I confirm that all methods were performed in accordance with the relevant guidelines. and informed consents were obtained from all participants.

### Questionnaire and data collection

The questionnaire was developed by drawing upon pertinent literature and established guidelines. Following the creation of the initial draft, feedback was solicited from two experts in cardiovascular disease and one gynecologist. A preliminary pilot study involving 64 participants was undertaken, yielding a Cronbach’s α coefficient of 0.780.

In our study, we employed a comprehensive questionnaire, presented in Chinese, which was structured into four dimensions. The basic information section included ten items designed to collect demographic and lifestyle data: Age, marital Status, residence, education, average monthly per capita income (RMB), average sleep hours per day, time to go to sleep, insomnia, and symptoms related to perimenopausal syndrome. Additionally, the questionnaire included 18 items for assessing knowledge, 10 items for attitude, and 10 items for practice. Within the knowledge dimension, correctness was scored 2 for an accurate response, 0 for an incorrect or unclear answer, resulting in a scoring range of 0 to 36 points. Attitude and practice items predominantly utilized a five-point Likert scale, spanning from very positive (5 points) to very negative (1 point). Consequently, the attitude dimension's score ranged from 10 to 50 points, and the practice dimension ranged from 10 to 50 points. Moreover, the study explored the Dysfunctional Beliefs and Attitudes about Sleep (DBAS) scale, comprising 16 items. Responses were graded from 0 for “strongly disagree” to 10 for “strongly agree,” yielding a total score range of 0 to 160 points. The average score of DBAS was computed for subsequent analysis. A total score equal to or exceeding 4 indicates incorrect or false beliefs about sleep, while a score below 4 signifies accurate or correct sleep beliefs^32^.

A stratified sampling survey methodology was employed, involving the distribution of a specific number of questionnaires in each district of Dezhou city, determined by population proportions. Participants were enlisted through diverse channels, including WeChat public accounts, WeChat groups, and hospital media. Data collection took place on the Wenjuanxing platform, with both offline and online questionnaires distributed concurrently. Offline questionnaires were dispensed in the Dezhou region of Shandong, with some collected in clinics at various hospitals and others randomly gathered on the streets of Dezhou city. Online questionnaires were disseminated through QR codes in WeChat groups or on official websites, targeting voluntary participants in the Dezhou region. Following the questionnaire distribution, follow-up verification occurred through phone calls. In cases where participants encountered issues during the survey, the research team members responsible for distributing the offline questionnaires addressed concerns, and contact information for team members handling inquiries was provided on the official website and WeChat group where the questionnaires were distributed.

For quality control, the research group comprises a total of seven team members, all of whom underwent uniform training before the project. The training covered the significance of questionnaire content, the aspects examined in each question, how to address participants' questions, and communication strategies. During offline questionnaire collection, team members assisted participants, provided prompt answers to queries, and ensured participants accurately understood the questions. Following the collection of online questionnaires, team members reviewed the gathered responses and ensured the quality of questionnaire completion through subsequent phone follow-ups.

### Sample size calcution

Sample size was calculated using the formula for cross-sectional studies: *α* = 0.05,${\text{ n}} = \left( {\frac{{Z_{1 - \alpha /2} }}{\delta }} \right)^{2} \times p \times \left( {1 - p} \right)$ where $Z_{1 - \alpha /2}$ = 1.96 when *α* = 0.05, the assumed degree of variability of p = 0.5 maximizes the required sample size, and δ is admissible error (which was 5%^33^). The theoretical sample size was 480 which includes an extra 20% to allow for subjects lost during the study.

### Statistical analysis

Statistical analyses were conducted using STATA 14.0 (STATA Corporation, College Station, TX, USA). Continuous variables were presented as mean and standard deviation. Comparisons between two groups were performed using t-tests, while ANOVA was employed for comparisons among multiple groups. Categorical variables were expressed as n (%). Pearson's correlation analysis was applied to examine the associations between knowledge (K), attitude (A), and practice (P). Multiple logistic regression analysis using the enter method was carried out with knowledge, attitude, and practice scores as dependent variables to identify independent risk factors for KAP scores. Knowledge, attitude, and practice scores reaching 70% or more of the theoretical total score were categorized as “sufficient knowledge,” “positive attitude,” and “proactive practice,” respectively. Variables with *P* < 0.05 in the univariate logistic regression were included in the multivariate regression analysis. Path analysis was utilized to explore the associations between knowledge, attitude, practice, and DBAS. Two-sided *P* < 0.05 was considered statistically significant.

### Ethics approval and consent to participate

The study was carried out after the protocol was approved by the Ethics Committee of Dongzhimen Hospital Affiliated to Beijing University of Chinese Medicine(2023DZMEC-174–01). I confirm that all methods were performed in accordance with the relevant guidelines. All procedures were performed in accordance with the ethical standards laid down in the 1964 Declaration of Helsinki and its later amendments, and informed consent was obtained from all participants.

### Supplementary Information

Supplementary Tables.

## Data Availability

All data generated or analysed during this study are included in this published article.
