# Supplementary material for: Knowledge, attitude, and practice toward sleep disorders and sleep hygiene among perimenopausal women
Source: Sci Rep. 2024 May 22;14:11663. doi: 10.1038/s41598-024-62502-4 (PMC11111451; doi:10.1038/s41598-024-62502-4)
Supplement: Supplementary file 1 — Supplementary Tables. [file 41598_2024_62502_MOESM1_ESM.docx]

**Supplementary Table 1.** DBAS-16 scores.

| **Variables** | **DBAS-16 consequences** | **P** | **DBAS-16 worry / helplessness** | **P** | **DBAS-16 expectations** | **P** | **DBAS-16 medication** | | **P** |  |
| --- | --- | --- | --- | --- | --- | --- | --- | --- | --- | --- |
|  | **Mean ± SD** |  | **Mean ± SD** |  | **Mean ± SD** |  | **Mean ± SD** | |  |  |
| **Marital Status** | | | | | | | | | |  |
| Single* | 8.22±1.61 | 0.519 | 6.72±1.51 | 0.942 | 8.75±1.64 | 0.521 | 6.36±2.49 | | 0.411 |  |
| Married | 7.87±2.10 |  | 6.74±1.75 |  | 8.82±2.01 |  | 6.78±2.53 | |  |  |
| **Residence** |  |  |  |  |  |  |  | |  |  |
| Rural | 7.89±2.03 | 0.715 | 6.74±1.72 | 0.531 | 8.67±1.95 | 0.009 | 6.89±2.50 | | 0.192 |  |
| Urban | 7.92±2.08 |  | 6.81±1.69 |  | 9.08±1.90 |  | 6.74±2.52 | |  |  |
| Suburban | 7.66±2.33 |  | 6.45±2.03 |  | 8.42±2.44 |  | 6.26±2.70 | |  |  |
| **Education** | | | | | | | | | |  |
| Primary school and below | 7.63±1.73 | 0.179 | 6.63±1.51 | 0.550 | 8.46±1.79 | 0.045 | 6.98±2.10 | | 0.789 |  |
| Junior high school | 7.93±2.06 |  | 6.80±1.76 |  | 8.78±1.99 |  | 6.74±2.56 | |  |  |
| High school/Technical secondary school | 7.95±2.23 |  | 6.63±1.91 |  | 8.96±2.05 |  | 6.82±2.67 | |  |  |
| College/bachelor's/master's degree | 7.86±2.16 |  | 6.80±1.68 |  | 8.95±2.03 |  | 6.60±2.58 | |  |  |
| **Average monthly per capita income (RMB)** | | | | | | | | | |  |
| < 2000 | 7.81±2.03 | 0.641 | 6.65±1.73 | 0.459 | 8.80±1.95 | 0.827 | 6.70±2.58 | | 0.668 |  |
| 2000-5000 | 7.89±2.06 |  | 6.73±1.74 |  | 8.86±1.99 |  | 6.73±2.48 | |  |  |
| 5000-10000 | 7.84±2.21 |  | 6.74±1.82 |  | 8.68±2.14 |  | 6.77±2.61 | |  |  |
| > 10000 | 8.20±1.95 |  | 7.07±1.60 |  | 9.06±1.64 |  | 7.18±2.49 | |  |  |
| **Time to go to sleep each day?** | | | | | | | | | | |
| Before 22:00 | 8.16±1.96 | 0.016 | 6.96±1.69 | 0.051 | 8.99±1.96 | 0.020 | | 7.06±2.54 | 0.045 | |
| 22:01-23:00 | 7.80±2.12 |  | 6.64±1.75 |  | 8.86±1.91 |  | | 6.63±2.57 |  | |
| After 23:00 | 7.51±2.16 |  | 6.54±1.83 |  | 8.32±2.22 |  | | 6.52±2.35 |  | |
| **Insomnia** | | | | | | | | | | |
| Yes | 7.76±2.01 | 0.104 | 6.74±1.66 | 0.987 | 8.54±1.98 | < 0.001 | | 6.70±2.41 | 0.452 | |
| No | 7.99±2.14 |  | 6.74±1.82 |  | 9.07±1.97 |  | | 6.82±2.64 |  | |
| **Symptoms related to perimenopausal syndrome** | | | | | | | | | | |
| Yes | 7.91±2.04 | 0.927 | 6.73±1.70 | 0.803 | 8.76±1.96 | 0.350 | | 6.65±2.41 | 0.165 | |
| No | 7.85±2.12 |  | 6.74±1.79 |  | 8.87±2.03 |  | | 6.87±2.64 |  | |

DBAS-16 reflected participants’ beliefs and attitude toward sleep in four different domains: (a) The consequences of insomnia (items 5, 7, 9, 12, and 16), (b) Worry/helplessness about sleep (items 3, 4, 8, 10, 11, and 14), (c) Sleep expectations (items 1 and 2), (d) Drugs (items 6, 13, and 15). * Single includes unmarried, divorced and widowed.

**Supplementary Table 2.** Knowledge dimension.

| **Knowledge, n (%)** | **Correct** | **Incorrect** | **Uncertain** |
| --- | --- | --- | --- |
| **Part 1** |  |  |  |
| 1. The main symptoms of sleep disorders include: |  |  |  |
| 1.1 Difficulty in falling asleep, taking longer than 30 minutes to fall asleep. | 414 (57.5) | 159 (22.08) | 147 (20.42) |
| 1.2 Decreased sleep quality, sleep maintenance disorders, ≥2 nocturnal awakenings, and early awakenings, etc. | 438 (60.83) | 161 (22.36) | 121 (16.81) |
| 1.3 Less than 8 hours of total sleep. | 453 (62.92) | 173 (24.03) | 94 (13.06) |
| 1.4 Daytime dysfunction, such as fatigue, decreased concentration, and unresponsiveness, etc. | 441 (61.25) | 163 (22.64) | 116 (16.11) |
| 1.5 Insomnia is a subjective experience; short sleep duration but no decrease in sleep quality cannot be regarded as insomnia. | 374 (51.94) | 178 (24.72) | 168 (23.33) |
| 2. Drinking alcohol before bedtime can effectively improve sleep quality. | 168 (23.33) | 312 (43.33) | 240 (33.33) |
| 3. Cognitive-behavioral therapy can improve clinical symptoms in insomnia patients without adverse reactions. | 279 (38.75) | 100 (13.89) | 341 (47.36) |
| 4. No matter what time you go to bed at night, getting 8 hours of sleep won't affect the quality of your sleep. | 210 (29.17) | 371 (51.53) | 139 (19.31) |
| **Part 2** |  |  |  |
| 1. Sleep disorders are one of the common problems in perimenopausal women and can manifest in a variety of ways, including not only insomnia, but also breathing and movement disorders. | 146 (20.28) | 322 (44.72) | 252 (35) |
| 1. Insomnia is categorized into short-term insomnia (< 3 months) and chronic insomnia (≥ 3 months) according to the duration of the disease. Some patients with short-term insomnia may be transformed into chronic insomnia due to misperception of sleep or inappropriate ways of self-regulation | 134 (18.61) | 320 (44.44) | 266 (36.94) |
| 1. Sleep-related movement disorders such as periodic leg movement disorder and restless leg syndrome significantly affect sleep, their symptoms worsen after menopause, and their treatment differs from that of insomnia disorders. | 104 (14.44) | 278 (38.61) | 338 (46.94) |
| 4. Do you know the five main components of cognitive-behavioral therapy for insomnia?  [Cognitive-behavioral therapy for insomnia mainly included: Sleep hygiene education, Relaxation therapy, Stimulus control, Sleep restriction, Cognitive therapy] | 99 (13.75) | 310 (43.06) | 311 (43.19) |
| 5. Do you know what is sleep hygiene education?  [Sleep hygiene education primarily helps insomnia patients understand the role of poor sleep habits in the onset and development of insomnia. Maintaining good sleep hygiene is a prerequisite for eliminating insomnia, but relying solely on sleep hygiene education is insufficient.] | 103 (14.31) | 283 (39.31) | 334 (46.39) |
| 6. Do you know what is relaxation training?  [Stress, tension and anxiety are common triggers for insomnia, and relaxation therapy can alleviate the adverse effects of these factors. Techniques to reduce arousal and promote nighttime sleep include progressive muscle relaxation, guided imagery and abdominal breathing exercises.] | 119 (16.53) | 315 (43.75) | 286 (39.72) |
| 7. What do you know about Stimulus Control Therapy?  [Mainly includes: 1) Only go to bed when sleepy; 2) If unable to fall asleep within 20 minutes, get out of bed and engage in simple activities until sleepy before returning to bed; 3) Avoid non-sleep-related activities in bed, such as eating, watching TV, listening to the radio, or pondering complex issues; 4) Maintain a regular wake-up time regardless of when you fall asleep.] | 131 (18.19) | 332 (46.11) | 257 (35.69) |
| 8. What do you know about sleep restriction therapy?  [1) Decrease bedtime to match actual sleep time, increasing bedtime by 15-20 min if sleep efficiency is maintained above 85% for at least 1 week; 2) Decrease bedtime by 15-20 min when sleep efficiency is below 80%; 3) Maintain bedtime unchanged when sleep efficiency is between 80% and 85%; and 4) Allow for regular naps not exceeding half an hour, avoiding other daytime naps, and maintaining a regular wake-up time].] | 113 (15.69) | 290 (40.28) | 317 (44.03) |
| What do you know about cognitive therapy?  [Mainly including: 1) maintain reasonable sleep expectations, do not blame all problems on insomnia; (2) maintain natural sleep, avoid excessive subjective intention to fall asleep (forcing oneself to fall asleep); (3) do not overly concerned about sleep, do not get frustrated because of a night of no sleep, develop tolerance to the effects of insomnia] | 120 (16.67) | 302 (41.94) | 298 (41.39) |
| 10. Research indicates that the optimal bedtime for adults is between 22:00-23:00, with an ideal sleep duration of 7-8 hours. | 252 (35) | 326 (45.28) | 142 (19.72) |

**Supplementary Table 3.** Attitude dimension.

| **Attitude, n (%)** | **Strongly Agree** | **Agree** | **Neutral** | **Disagree** | **Strongly Disagree** |
| --- | --- | --- | --- | --- | --- |
| 1. I think perimenopausal sleep disorders are quite normal and do not need special treatment, the symptoms will disappear automatically after this period. (N) | 82 (11.39) | 204 (28.33) | 266 (36.94) | 121 (16.81) | 47 (6.53) |
| 2. Sleep disorders make me feel anxious and uneasy. (N) | 193 (26.81) | 277 (38.47) | 206 (28.61) | 32 (4.44) | 12 (1.67) |
| 3. Nocturnal sleep disorders seriously interfere with my daytime life. (N) | 245 (34.03) | 276 (38.33) | 153 (21.25) | 38 (5.28) | 8 (1.11) |
| 1. When I don't sleep well the night before, I feel like terrible the next day. (N) | 266 (36.94) | 260 (36.11) | 152 (21.11) | 32 (4.44) | 10 (1.39) |
| 5. When insomnia is severe, seeking help from professionals is necessary, rather than enduring it. (P) | 311 (43.19) | 222 (30.83) | 164 (22.78) | 15 (2.08) | 8 (1.11) |
| 6. Creating a comfortable sleep environment can help improve sleep quality. (P) | 339 (47.08) | 232 (32.22) | 129 (17.92) | 13 (1.81) | 7 (0.97) |
| 7. Measures like muscle relaxation, deep breathing, and meditation can help improve sleep quality. (P) | 282 (39.17) | 245 (34.03) | 161 (22.36) | 22 (3.06) | 10 (1.39) |
| 8. When insomnia is severe, I would consider taking medication to assist with sleep. | 170 (23.61) | 258 (35.83) | 185 (25.69) | 76 (10.56) | 31 (4.31) |
| 9. Regular psychological counseling and maintaining a relaxed and cheerful mood can be helpful in alleviating sleep disorders. (P) | 254 (35.28) | 273 (37.92) | 162 (22.5) | 21 (2.92) | 10 (1.39) |
| 10. If there are educational lectures on perimenopausal sleep disorders, I would be very willing to participate. (P) | 253 (35.14) | 237 (32.92) | 199 (27.64) | 22 (3.06) | 9 (1.25) |

“N” means negative; “P” means positive.

**Supplementary Table 4.** Practice dimension.

| **Practice, n (%)** | **Always** | **Often** | **Sometimes** | **Rarely** | **Never** |
| --- | --- | --- | --- | --- | --- |
| 1. My daily schedule is quite regular, with fixed times for both bedtime and waking up. (P) | 248 (34.44) | 215 (29.86) | 149 (20.69) | 66 (9.17) | 42 (5.83) |
| 2. I would have a drink before bedtime to help myself fall asleep. (N) | 31 (4.31) | 30 (4.17) | 103 (14.31) | 129 (17.92) | 427 (59.31) |
| 3. I rely on taking sleeping pills every day to help myself fall asleep. (N) | 37 (5.14) | 48 (6.67) | 98 (13.61) | 111 (15.42) | 426 (59.17) |
| 4. I deliberately increase daytime physical activity to improve the depth and quality of nighttime sleep. (P) | 58 (8.06) | 74 (10.28) | 231 (32.08) | 181 (25.14) | 176 (24.44) |
| 5. Before going to bed, I deliberately create a comfortable sleep environment, such as temperature and lighting. (P) | 96 (13.33) | 117 (16.25) | 193 (26.81) | 161 (22.36) | 153 (21.25) |
| 6. Before going to bed, I will play soothing music to adjust the mood and make it easy to fall asleep. (P) | 56 (7.78) | 75 (10.42) | 209 (29.03) | 152 (21.11) | 228 (31.67) |
| 7. Before bedtime, I engage in stimulating activities such as playing cards, mahjong, gatherings, or watching exciting movies. (N) | 30 (4.17) | 33 (4.58) | 99 (13.75) | 136 (18.89) | 422 (58.61) |
| 8. I have a habit of contemplating complex work or life issues before bedtime. (N) | 46 (6.39) | 64 (8.89) | 198 (27.5) | 174 (24.17) | 238 (33.06) |
| 9. When experiencing insomnia, I seek out family or friends to chat and share my feelings. (P) | 44 (6.11) | 54 (7.5) | 194 (26.94) | 174 (24.17) | 254 (35.28) |
| 10. When insomnia problems become severe, I will actively seek medical advice from a doctor. (P) | 92 (12.78) | 77 (10.69) | 236 (32.78) | 157 (21.81) | 158 (21.94) |

“N” means negative; “P” means positive.
